# Editorial: Lung adenocarcinoma: from genomics to immunotherapy

**DOI:** 10.3389/fgene.2024.1399127

**Published:** 2024-04-09

**Authors:** Yiming Meng, Maria Palmieri, Elisa Frullanti

**Affiliations:** ^1^ Department of Central Laboratory, Cancer Hospital of Dalian University of Technology, Liaoning Cancer Hospital and Institute, Shenyang, China; ^2^ Cancer Genomics and Systems Biology Lab, Department of Medical Biotechnologies, University of Siena, Siena, Italy; ^3^ Department of Medical Biotechnologies, Med Biotech Hub and Competence Centre, University of Siena, Siena, Italy

**Keywords:** solid cancer, cancer biomarkers, cancer prognosis, disease monitoring, lung cancer, precision medicine

## Introduction

Lung cancer is the second most common type of cancer and is the leading cause of cancer death globally. In 2018, almost 2.1 million new cases were diagnosed, accounting for ∼12% of the cancer burden worldwide (*Sung et al., 2021*). The malignant stage of lung cancer is known as lung adenocarcinoma, which is the most common and is diagnosed in both smokers and non-smokers.

There are two main types of lung cancer, the non-small cell lung cancer (NSCLC) and the small cell lung cancer (SCLC). Genomic studies have indicated that more than 80% of lung malignancies are classified as NSCLC, of which adenocarcinoma is the predominant subtype. In metastatic patients, although significant progress has been made for tumors harboring druggable mutations such as EGFR, the majority of those is lacking of such mutations and the prognosis remains poor. Platinum doublet chemotherapy has been the mainstay first-line treatment for patients who are diagnosed with metastatic lung adenocarcinoma without a targetable mutation (*Bodor et al., 2018*).

In recent years, immunotherapy has emerged as a treatment option that has shown a strong response in a subset of patients. The immune agents block crucial checkpoints and regulate the immune response, but the tumor cells evade the patient’s immune system. By blocking these receptor–ligand interactions, a particular subset of T cells is activated to recognize and respond to tumor cells. While such responses to immunotherapy are promising, they have only been effective in ∼20% of patients (*Murciano-Goroff et al., 2020*).

Therefore, there is an urgent need to understand the underlying mechanism of lung adenocarcinoma from genome to immunotherapy. To address this unmet need, this Research Topic will focus on advancements related to lung adenocarcinoma (LUAD) and the identification of novel biomarkers as new therapy-determining or companion prognostic tools for the development of precise mechanism-based treatments.

## Novel prognostic biomarkers for lung adenocarcinoma

The original articles published in the present Research Topic updated about novel prognostic biomarkers in lung adenocarcinoma patients through *in silico* approaches. In particular, Wang et al. F’s group assessed the roles of unlocking phenotypic plasticity (UPP) in immune status, prognosis, and treatment in patients with LUAD based on the cancer genome atlas (TCGA) database (https://www.frontiersin.org/journals/genetics/articles/10.3389/fgene.2022.941567/full). They proposed UPP as a new and reliable prognosis indicator to predict the patient’s overall survival and help the clinician to predict therapeutic responses and make individualized treatment plans.

Similarly, Zhou X et al. investigated the expression of indolethylamine N-methyltransferase (INMT) and its clinical value as a prognostic biomarker in LUAD based on TCGA and Gene Expression Omnibus (GEO) databases (https://www.frontiersin.org/journals/genetics/articles/10.3389/fgene.2022.946848/full). They found that INMT expression was significantly downregulated in LUAD, and the low expression of INMT was associated with poor prognosis but favorable immunotherapy response in LUAD.

Song Y et al. highlighted the association of necroptosis with LUAD and its potential use in guiding immunotherapy based on transcriptomic and clinical data of patients from TCGA and GEO databases (https://www.frontiersin.org/journals/genetics/articles/10.3389/fgene.2022.1027741/full). They analyzed 902 samples and identified a prognostic signature of five necroptosis-related genes that could be used to predict the prognosis of LUAD patients.

Additionally, Zhu X’s group focused their attention on the role of basement membranes (BMs) and their related genes for prognosis prediction in LUAD patients from TCGA and GEO databases (https://www.frontiersin.org/journals/genetics/articles/10.3389/fgene.2023.1100560/full). They used a training set of data and a verification cohort and identified a prognostic signature of ten BM-associated genes that could be used to predict the prognosis of LUAD patients and guide personalized treatment.

Zhang et al. investigated the relationship between cuproptosis and long non-coding RNAs (lncRNAs) in carcinogenesis and prognosis/treatment of LUAD patients based on transcriptomic data of 507 samples from TCGA database (https://www.frontiersin.org/journals/pharmacology/articles/10.3389/fphar.2023.1236655/full). They constructed a prognostic model associated with the prognosis of patients with LUAD undergoing therapy and confirmed their results through *in-vitro* experiments.

Finally, Liu R’s group has dedicated its work to studying the correlation between neutrophils and tumor development in LUAD based on data from the TCGA database and *in-vitro* experiments, identifying 30 hub genes that were significantly associated with neutrophil infiltration and developing a neutrophil scoring system associated with prognosis, and tumor immune microenvironment.

## Relevant case reports

The present Research Topic also contains interesting, unusual, and noteworthy case reports that can help clinicians and scientists identify new trends, evaluate new therapeutic effects, as well as create new research questions. In particular, Hodges A et al. presented a 62-year female with Lynch syndrome, who developed an EGFR-positive lung adenocarcinoma highlighting the complex interplay of genetic cancer predisposition syndromes and the development of spontaneous driver mutations in the disease course and the subsequent management of tumors arising (https://www.frontiersin.org/journals/oncology/articles/10.3389/fonc.2023.1193503/full).

Li H et al. presented a 35-year female with a rare lung cancer exhibiting choriocarcinoma features demonstrating the potential of chemo-immunotherapy in treating this aggressive subtype of lung cancer (https://www.frontiersin.org/journals/oncology/articles/10.3389/fonc.2024.1324057/abstract).

Last but not least, Quanqing L et al. presented a 67-year female with a squamous cell carcinoma (NSCLC) that transforms into small cell carcinoma (SCLC) after five cycles of immunotherapy targeting PD-1 treatment (Sintilimab) of NSCLC (https://www.frontiersin.org/journals/oncology/articles/10.3389/fonc.2024.1329152/full). This histological transformation could represent a potential mechanism of cancer therapeutic resistance.

## Conclusion

In conclusion, this Research Topic highlights the importance of good prognostic biomarkers in determining the most effective treatment and revolutionizing cancer precision medicine. The Research Topic of articles provides a comprehensive overview of current advancements in prognostic and therapeutic lung cancer biomarkers offering a substantive framework that informs ongoing scientific inquiry and clinical practice, aiming to improve the understanding and management of LUAD patients.
